# Complete genome sequence of *Pediococcus pentosaceus* 13.7 2A-1 isolated from a Holstein Friesian dairy cattle

**DOI:** 10.1128/mra.00848-25

**Published:** 2025-10-02

**Authors:** Nadine Mariani Corea, Fritz Titgemeyer, Jennifer Wachtarczyk, Frank Meyer, Sebastian W. Fischer

**Affiliations:** 1 Department of Food, Nutrition, Facilities, FH Münster 237055 , Münster, Germany; 2 Institute for Hygiene and Public Health, University Clinics Bonn, Bonn, Germany; Nanchang University, Nanchang, Jiangxi, China

**Keywords:** lactic acid bacteria, probiotics, protective cultures, nanopore, biofilm, food, food safety

## Abstract

*Pediococcus pentosaceus* was isolated from a Holstein Friesian dairy cattle from a conventionally operated farm. The complete genome of strain *P. pentosaceus* 13.7 2A-1 comprises 1.84 Mbp and 1,746 protein-coding sequences. The decoded genome sequence will serve to further study the strain for its use in food fermentation and food safety.

## ANNOUNCEMENT

Strains of *Pediococcus pentosaceus* are used for food fermentation and preservation (1, 2). We aim to isolate *P. pentosaceus* strains from natural environments to describe their efficacy against pathogenic, food-borne microorganisms (3, 4).

*P. pentosaceus* 13.7. 2A-1 was isolated from a Holstein Friesian dairy herd on a conventionally managed farm in Münsterland, Germany. The teat canal biofilm was collected from the teat canal using a swab and transferred to a 0.9% NaCl solution. Dilutions were plated onto De Man, Rogosa, and Sharpe (MRS)-Bouillon-agar containing 0.5 g/L cysteine and 10 mg/L bromophenol blue and incubated anaerobically at 30°C for 48 h (5). A single colony was subjected to polymerase chain reaction using primers 27f (5′-AGAGTTTGATCCTGGCTCAG-3′) and the self-designed N1492r, in which positions 6 and 17 were replaced by Y bases (5′-TACGGYTACCTTGTTAYGACTT-3′) compared to the original universal primer 1492R (6). The Sanger-sequenced DNA of 1,067 bp was 100% identical to positions 66 to 1,133 of *rrnA* of type strain *P. pentosaceus* ATCC 25745 (NC_008525.1) (7).

Genomic DNA was isolated using the Wizard HMW DNA Extraction Kit (Promega, USA) and quantified on a DeNovix QFX fluorometer (DeNovix, USA). Two independent libraries were prepared with the Oxford Nanopore SQK-RAD004 kit. Sequence data were collected by two independent nanopore runs using Flongle R9.4.1 Flow Cells. Basecalling was performed with Guppy v.6.3.8 (model dna_r9.4.1_450bps_hac.cfg), retaining reads with a quality score ≥Q9 (8). Adapters were trimmed using Porechop v0.2.4. Filtlong v0.2.1 discarded the lowest 15% of reads and <1 kb. (9, 10). The data set for genome assembly was characterized with Nanoq v.0.10.0 and yielded 28,792 reads (131.9 Mbp; N50 = 5,081 bp; median/avg = 3,284/4,580 bp; max = 86,128 bp; mean Q = 9.8). The quality metrics of 30.8%/6.1%≥Q20/Q30 were calculated with SeqKit v.2.9.0 (11, 12). The genome was assembled using Flye v.2.9.2, Raven v.1.8.2, and Miniasm v.0.3 + MiniPolish v.0.1.3 implemented in the Trycycler v.0.5.4 pipeline (13–17). The draft was polished and verified (Medaka v.1.8.0; Bandage v.0.9.0) (18, 19). FAST5 reads were re-basecalled for final polishing (Dorado v.0.9.6; model dna_r9.4.1_e8_sup@v3.6 with foundation), then aligned (Dorado Aligner), and filtered (Samtools v.1.22.1; MAPQ ≥20; flags QC-failed 0×200, secondary 0×100, and supplementary 0×800 excluded; ≥1 kb) (20, 21). The genome was polished with Medaka v.2.1.0 and Homopolish v.0.4.1 (polish, modpolish) and reoriented to *dnaA* as the first base (Dnaapler v.1.2.0) (18, 22, 23). Unless otherwise noted, all bioinformatics tools were used with default settings.

The genome of type strain *P. pentosaceus* ATCC 25745 (NC_008525.1) was taken as reference to obtain genome length, G + C content, and percentage of overlap (genome fraction) using QUAST v.5.3.0 (Table 1). The read depth, determined by reassigning the filtered reads, yielded a coverage of 51.7× across the entire chromosome (minimap2 v.2.30; samtools v.1.22.1) (21, 24–26). Genome completeness was inferred with BUSCO v.5.8.3 (data set *pediococcus_odb12*) and CheckM2 v.1.1.0 (neural-network model) (27, 28). The genome was annotated with PGAP v.6.10, identifying 1,746 protein-coding sequences, 15 rRNA genes, and 55 tRNA genes (29).

**TABLE 1:** Assembly and quality metrics for the complete chromosome of *P. pentosaceus* 13.7 2 A-1

Replicon	Length (bp)	G + C (%)	Contigs coverage	BUSCO genome completeness (%)	CheckM2 genome completeness and contamination (%)	Genome fraction (%)
Chromosome	1,836,247	37.1	51.7×	97.8	100/0.18	87.9

## Data Availability

Whole-genome sequencing data for *P. pentosaceus* isolate 13.7 2 A-1 have been deposited in the NCBI Sequence Read Archive (SRA) under BioProject accession number PRJNA1297324. The raw sequencing reads are available via the SRA under the corresponding BioSample SAMN50231305 entry. The assembled and annotated genome is available at DDBJ/ENA/GenBank accession numbers CP197205 and GCA_052059775.1.
